# Relational community engagement within health interventions at varied outcome scales

**DOI:** 10.1371/journal.pgph.0003193

**Published:** 2024-06-11

**Authors:** Nicole Redvers, Asiya Odugleh-Kolev, Joanna Paula Cordero, Felicia Zerwas, Natalie Mariam Zitoun, Yasaman Mohammadi Kamalabadi, Amy Stevens, Ahimza Nagasivam, Paul Cheh, Emma Callon, Katthyana Aparicio-Reyes, Shogo Kubota

**Affiliations:** 1 Schulich School of Medicine and Dentistry, University of Western Ontario, London, Canada; 2 Department of Integrated Health Services, World Health Organization, Geneva, Switzerland; 3 Health Promotion and Social Determinants of Health Unit (HPD), World Health Organization African Regional Office, Brazzaville, Republic of the Congo; 4 Department of Psychology, New York University, New York, New York, United States of America; 5 School of Public Health, Yorkshire and the Humber Postgraduate Deanery, Leeds, United Kingdom; 6 School of Public Health, Health Education England, London, United Kingdom; 7 The Equity Initiative, China Medical Board Foundation, Bangkok, Thailand; 8 Division of Healthy Environments and Populations, World Health Organization Western Pacific Regional Office, Manila, Philippines; 9 Maternal Child Health and Quality Safety, World Health Organization Western Pacific Regional Office, Manila, Philippines; Emory University, UNITED STATES

## Abstract

Relational community engagement may be a powerful approach with multiple health outcomes. Relational community engagement has the potential to promote health and involves collaborative efforts between multiple stakeholders. The COVID-19 pandemic further highlighted the centrality of community engagement in health crises. Challenges continue to persist, however, in genuinely engaging and empowering communities for better health outcomes. Understanding the multi-level and complex relational nature of community engagement is essential to comprehend its influence on health at micro, meso, and macro scales of influence. The purpose of this narrative review was to synthesize the literature on relational community engagement within varied health interventions at the three major system levels (micro, meso, and macro) to support the development of future research agendas. At the micro level, relational community engagement interventions demonstrated a range of positive outcomes including: increased sense of control, satisfaction, positive behavior, improved knowledge, behavior change, empowerment, and overall positive health and social outcomes. At the meso level, relational community engagement interventions resulted in increased trust between stakeholders and groups/teams, and increased community senses of ownership of interventions, decisions, structures. At the macro level, relational community engagement interventions influenced broader societal factors and had positive impacts on health policy and governance including collaboration between sectors and communities as well as increased access to services. The review highlights the potential versatility and effectiveness of interventions that prioritize relationships, health promotion, and social change while underscoring the significance of holistic and community-centered approaches in addressing diverse health and social challenges.

## Background

Community engagement is an approach aimed at addressing health-related issues, promoting well-being, and acting on the social determinants of health. Community engagement has been defined by the World Health Organization (WHO) as “a process of developing and maintaining relationships that enable stakeholders to work together to address health-related issues and promote well-being to achieve positive and sustainable health impact and outcomes” [1]. Community engagement involves establishing trust-based relationships and collaborating to develop more effective health interventions, programs, services, and policies while empowering communities as key participants in health initiatives—ultimately leading to positive and sustainable health outcomes [2–4]. The approach helps uncover and tackle local issues, implement grassroots solutions, and leverage local resources and networks to sustain health interventions and outcomes [5].

People and communities are vital for promoting overall health and well-being, with their active involvement being crucial for integrated health services and person-centered care. The WHO has also encouraged broadening the definition of “community” to acknowledge the multiple interconnected communities that cross time and space (e.g., families, schools, neighborhoods, and places of work; early childhood, adulthood) that shape individual and collective identity, choices, and behaviors [6]. Additionally, governments have increasingly recognized the significance of collaborating with communities across various sectors to deliver a wide range of services [7]. The COVID-19 pandemic, however, exposed the vulnerabilities of populations and underscored the need for ongoing and effective community engagement for improved health outcomes. These include addressing historical mistrust in government and healthcare, ensuring community engagement strategies consider the unique needs, cultural contexts, and communication preferences of vulnerable populations while not only relying on digital platforms. Despite the longstanding concepts of "community" and "community engagement," building high-quality services and systems that genuinely engage and empower people remains a persistent challenge, as evidenced by the response to the pandemic [3, 8].

The WHO has been actively supporting research efforts to better understand the benefits and mechanisms of community engagement with the aim of enhancing the overall quality of care for people and communities [9–11]. The emerging evidence emphasizes a holistic "whole person-whole system" approach to community engagement, recognizing it as a social process encompassing physical, emotional, mental, social, and spiritual dimensions [3]. This departs from historical views of community engagement processes which have often been viewed as a very linear process of engagement without an appreciation of the complexities often inherent in relationship building for successful outcomes (e.g., power differentials). Furthermore, the recognition and resurgence of acknowledging the ‘relational’ components within community engagement have been underappreciated in wider health system circles [12, 13].

Achieving successful community engagement often requires an understanding of the attributes and nature of human “relationships,” including trust, resilience, and responsiveness [14]. The strength of relational connections between individuals and their sense of safety within their communities may be significant indicators of success. Additionally, health systems can also be considered as social systems with a complex network of relationships that impact trust and system performance [15, 16]. Consequently, the relationality of community engagement may be a powerful lever and mechanism to support the journey toward establishing a culture of quality and compassion in healthcare settings [17, 18].

The understanding of the importance of relational connections for well-being has been well appreciated within Indigenous communities where ‘relationality’ is a fundamental tenet of community existence and function [19]. Indigenous communities can see relationality as a state where one experiences the self as a part of others, and that others are part of the self—all are inextricably linked [20]. Understanding and operating within a relational existence over time becomes central as it may profoundly influence service performance, satisfaction, and ultimately, health outcomes. Ultimately, a well-connected, purposeful, and aligned healthcare system characterized by trust, resilience, and responsiveness is essential in the journey toward achieving high-quality primary healthcare and universal health coverage [3]. Therefore, an understanding of the key facets of community engagement has led to renewed interest and use in what we are calling ‘relational community engagement’ which bridges understandings of the ‘relationality’ already inherent within the concept of ‘community engagement’.

**Fig 1:**
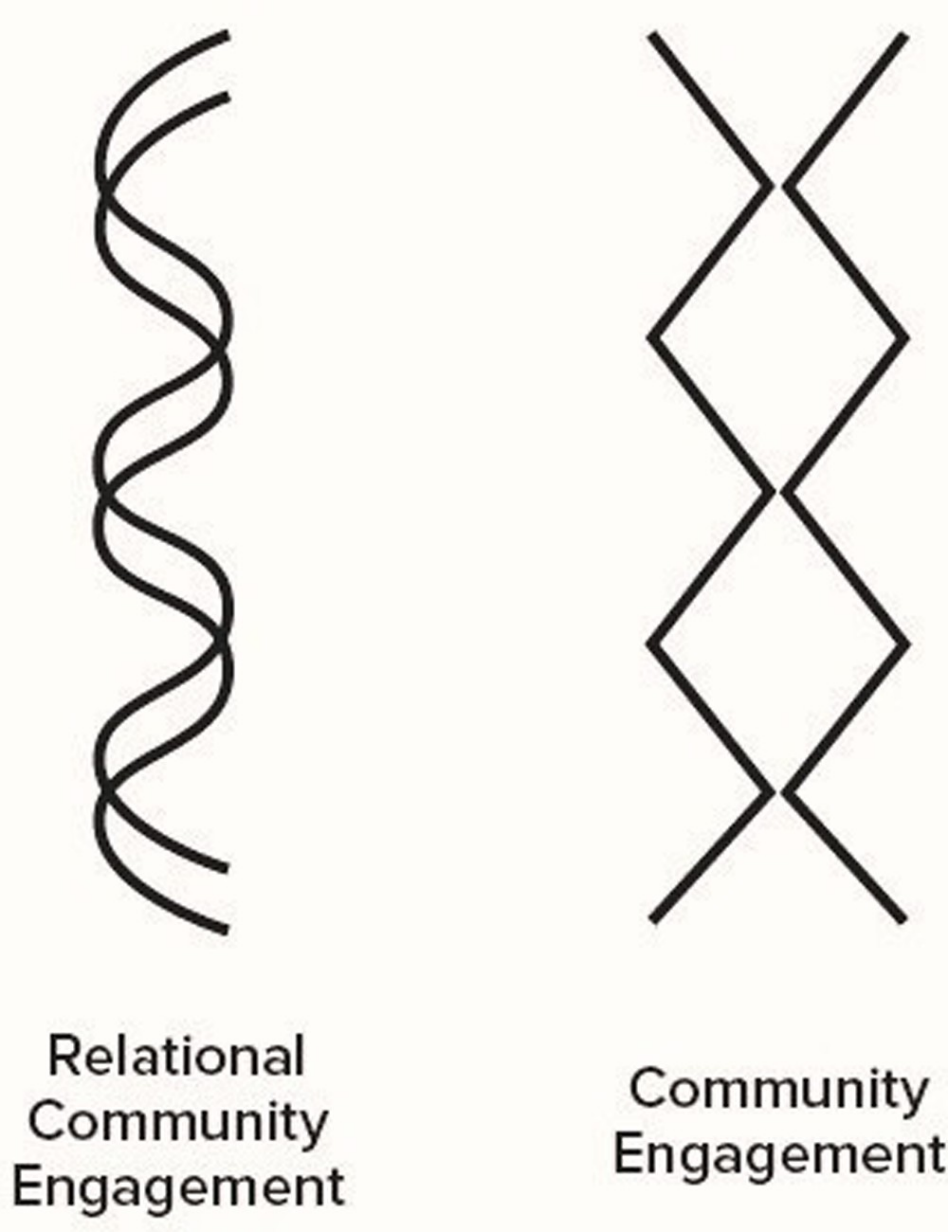
Our depiction of relational community engagement (i.e., reciprocal and interconnected exchange that is ongoing) versus commonly practiced community engagement (i.e., transactional, touch point only exchange).

We describe ‘relational community engagement’ as an approach that conjoins individual and collective awareness, and is intentional about processes that facilitate positive connection, belonging, and communication—all of which are needed for meaningful collaboration and co-production. This approach emphasizes and centers on building nurturing, ongoing, and longstanding relationships between different stakeholders, including community members, organizations, and institutions for improved health outcomes (see *Fig 1*). Rather than focusing solely on specific projects or outcomes, relational community engagement places a strong emphasis on developing and maintaining long-term connections and trust within communities [2, 21–24]. Our relational community engagement approach has been developed alongside other relational community engagement efforts that we wish to acknowledge (e.g., *Vanasupa & Schlemer*, *2014* [25], which focuses on relational community engagement having a large scope of shared aspirations, collaborative co-creating that is emergent in nature, has social value, and has an emphasis on developing thriving communities).

In recent years, the significance of relational community engagement in shaping health outcomes has garnered increasing attention as a multifaceted and intricate phenomenon [26–29]. There is a need, however, to better clarify the multi-level and complex nature of relational community engagement to better understand its influence on health at different levels of impact (see *Table 1*). This narrative review therefore sought to synthesize the multi-level and complex nature of relational community engagement and its profound influence on health at different scales (i.e., micro, meso, and macro). The review was intended to be a high-level “snapshot” view of the literature to support the development of future research needs in the area of relational community engagement. A narrative review approach was engaged due to its ability to consider variation in the formats of included information while permitting the generation of a wider and more inclusive picture of available resources given the nature of the topic [30].

**Table 1:** Complexity of relationality (reproduced from WHO with permission*) [3].

Dimension of Relationality	Levels of Analysis	Site of Impact of Change	Potential Health Outcomes
Complexity of individual interactions/relations	Micro	Persons	• Improved sense of well-being • Increased (individual) sense of control • Increased (individual) sense of empowerment • Increased (individual) sense of ownership of interventions
Complexity of group interactions/relations	Meso	Communities, Teams, Groups	• Improved health status of communities • Better reach of health interventions (e.g. to vulnerable or marginalized communities) • Increased trust between stakeholders, groups, teams • Co-design of interventions
Complexity of whole-system interactions/relations	Macro	Programmes, Systems, Organizations	• Healthier policies (e.g., health-affirming, well-being promoting) • Decreased health inequities • Equitable dynamic flow of power, control and resources within and across all levels of programming, systems, and organizations

*From: Evaluation of the WHO community engagement research initiative. Manila: World Health Organization

Regional Office for the Western Pacific; 2023. License: CC BY-NC-SA 3.0 IGO.

### Types of relational community engagement interventions

Table 2 presents a list of relational community engagement interventions along with their corresponding definitions. Among the varying relational community engagement interventions currently being engaged within health spaces, improving accountability and governance as well as promoting social participation are often highlighted as outcomes. More specifically, there are intervention efforts emphasizing the importance of transparency and responsibility through relationships [31], while recognizing the significance of involving individuals and communities in decision-making processes [32]. Capacity building among healthcare providers (i.e., aiming to enhance their knowledge and skills), as well as interventions to improve the quality of services while emphasizing the delivery of high-standard care have also been carried out within relational settings [33]. Interventions have been focused less commonly on community engagement as an ‘outcome’ (i.e., explicitly recognizing its value in health-related initiatives) [34]. Other kinds of relevant interventions include: targeted interventions to reduce health and social inequities while addressing disparities in healthcare access [35]; implementing health promotion interventions while emphasizing preventive measures and health education [36]; improving access to services by removing barriers; and, empowering health seekers by encouraging active participation in managing their health [33]. Mixed intervention methods (i.e., implementing varied combinations of the different intervention types outlined in *Table 2*) with a comprehensive approach that combines multiple strategies (e.g., improving access to and quality of services) appear to be employed more often within relational community engagement interventions. Additionally, relational community engagement interventions often embody multiple categories of interventions (i.e., they are multi-layered, incorporating different levels of interventions) [33].

**Table 2:** Example interventions.

Intervention	Definition
**Promoting social participation**	Interventions that aim to foster increased social interactions and cultivate a sense of community or belonging. These interventions target community members with limited social interactions (e.g., older adults) or those facing challenges in interacting with others (e.g., individuals living with communication disabilities or those recovering from trauma).
**Health promotion**	Activities and programs that are designed to encourage healthy lifestyles and prevent illnesses. They may either focus on specific health issues or take a holistic approach addressing multiple causes of ill-health within a community.
**Capacity building among health workers**	Interventions concentrating on training programs or collaborative learning for health workers to enhance their communication skills, respectfulness toward colleagues and patients, cultural sensitivity, and understanding of social determinants of health.
**Improving quality of services**	Quality improvement interventions, often incorporating collaborative elements, to ensure that services (e.g., primary healthcare, pediatric care, family planning services) meet high standards. These interventions are frequently combined with other actions.
**Increasing community engagement**	Interventions encouraging the participation and involvement of community members in service provision or health promotion. They seek to empower community members to address barriers to quality services and volunteer to extend service accessibility. These activities are often integrated with other intervention types.
**Empowering health seekers**	Interventions aimed at supporting health seekers to communicate effectively with health workers and enhance their preparedness and ability to safeguard their health and well-being. This empowerment is typically complemented by other interventions.
**Improving access to services**	Interventions focusing on expanding service reach while addressing access barriers such as cost and geographical distance. Efforts are directed toward ensuring that hard-to-reach and marginalized communities receive adequate attention.
**Improving accountability and governance**	Interventions including community monitoring, social accountability measures, and health facility management/health committees. These mechanisms enable community members to monitor and hold health workers and authorities accountable for the services provided.
**Reducing health and social inequities**	Interventions targeting the reduction of disparities, including social determinants of health and marginalization, which render certain groups more vulnerable to diseases or at-risk factors. This includes efforts to address unequal access to services.

### Relational community engagement intervention outcomes at micro, meso, and macro levels

#### Micro level

Overall, at the micro level, relational community engagement has assumed a focal point for individual behaviors and health outcomes. Here, the emphasis has lied in comprehending and addressing the unique needs and perspectives of individuals within a community. The personalized approach recognizes that every individual’s well-being is integral to the overall health of the community as a whole. By acknowledging the diverse backgrounds, preferences, and challenges faced by community members, targeted interventions are designed to promote positive health behaviors and tailor support to foster a healthier collective [3, 24, 37].

For example, in an article by Alexander et al. [38], it was found that respondents viewed a relational multi-stakeholder alliance (i.e., community partnerships) as having a clear and shared vision of health in their communities (89%), and that the alliance was taking meaningful actions (83%) to improve individual and community health. The benefits of participating in the alliance was found to outweigh the costs of participation for individuals (88%). In another article by Bailey et al. [34], they observed that participants in community development initiatives felt empowered through knowledge and social connection. Families had a better understanding of available services and were more empowered to request them. The operation teams were clear about the roles of service providers, and community members became empowered through their knowledge of the processes. Baur et al. [39] found that community development efforts resulted in an increased sense of belonging and new friendships and relationships among residents [39]. Residents also felt proud and enthusiastic about the successful outcomes from the community gatherings they hosted. Gerber et al. [32] additionally highlighted the unexpected outcome of increased growth of Centers for Disease Control and Prevention (CDC) personnel in working collaboratively with Tribal communities in the United States. Staff improved their knowledge and skills for engaging Tribes appropriately and reported feeling more capable of advocating for systems change, implementing public health improvements, and integrating cultural knowledge into their efforts with better relationships. Finally, in an article by Rosa Hernandez et al. [40] they highlighted that Interactive Group Play (IGP) provided an opportunity for reminiscing and facilitated moments of learning, leading to meaningful relationships being formed.

In summary, at the micro level, the literature consistently demonstrates positive outcomes in knowledge improvement [39, 41], behavior change [42], increased empowerment [36, 43], strengthened community engagement [32, 44], and improved health and social outcomes resulting from community development [45], health promotion [32], and social change interventions [46]. These findings collectively indicated the potential of varied ‘relational’ community engagement approaches in fostering positive micro-level outcomes across the diverse communities.

#### Meso level

At the meso level, the focus of relational community engagement often shifts toward understanding the interconnectivity of various community subgroups. The meso level encompasses organizations, communities, and populations, while considering factors such as social networks, community resources, and healthcare facilities. Relational community engagement at the meso level often involves collaboration and interventions targeting specific groups or communities. The dynamics of relationships at this level appears to play a pivotal role in shaping health-related policies, services, and initiatives within the communities noted. By fostering collaborative partnerships and leveraging existing resources, a more integrated and holistic approach to community health may be achieved [3, 37, 47, 48].

For example, Alexander et al. [38] evaluated the impact of the Aligning Forces for Quality (AF4Q) initiative, which resulted in the creation of a network of communities providing mutual support for programmatic interventions to improve healthcare quality and value. This networking and relationship-based approach led to an increase in learning opportunities and the diffusion of innovations and best practices that could be adapted to local needs and conditions. Bravo et al. [49] documented the formation and expansion of a regional collaboration to increase the use of chronic prevention services among an underserved aging community. This collaboration led to increased community health centers (CHC) and NGO capacity, and forged CHC-community linkages to promote health. Another article by McEvoy et al. [50] highlighted how the Eis Ledader project increased the number of Jewish residents accessing the Improving Access to Psychological Therapies (IAPT) service, fostered relationships, and built collaborative partnerships with cultural resources. Finally, an article by Ståhl et al. [51] found that the development of trusting relationships between participants and officials resulted in more realistic recovery and rehabilitation plans.

In summary at the meso level, the literature demonstrates the effectiveness of various relational community engagement interventions in achieving positive outcomes, including increased community awareness of various health issues (e.g., HIV transmission) [41], improved healthcare quality [52], enhanced community cohesion [53, 54], strengthened relationships with authorities and service providers [36, 55], and increased access to services and resources [56, 57]. The success of these interventions emphasizes the importance of community involvement and collaboration in addressing various health and social challenges.

#### Macro level

At the macro level, the focus of relational community engagement tends to correspond to the broader societal context. Here, the collective efforts of engaged communities influence public health policies, social determinants of health, and ultimately contribute to larger systemic changes. Relational community engagement at this level examines broader societal factors, including the social, economic, and political determinants of health. Relational community engagement approaches emphasize policy changes, advocacy efforts, and large-scale interventions aimed at improving population health and addressing social inequalities [3, 58].

For example, the Community-Based Organizations (CBOs) and Civil Society Organizations (CSOs) played a crucial role in raising HIV risk perception among community members, facilitated by cooperation and relationship building between local council members and different actors [41]. An article by Bailey et al. [34] additionally demonstrated community interventions like Operation RESET (an initiative to improve the identification and prosecution of child sexual abuse incidents in remote Indigenous communities) led to increased interagency coordination and collaboration, thereby leading to improved child sexual abuse reporting and interagency cooperation. Collaborative approaches, such as community health-center partnerships with stakeholders, also positively impacted the success of obesity intervention programs [59]. Technical assistance provided by another project resulted in substantial improvements in immunization, skilled birth attendance, facility deliveries, and sanitary latrines within intervention areas [43]. Engaging various stakeholders, including community representatives, in supporting local health facilities led to shared ownership of problems and solutions, improving the quality of care in maternal and neonatal health in another study [57]. Finally, relationship-based participatory activities and the establishment of action plans improved community organization and coordination with government and NGO services in an article by Pridmore et al. [35].

In summary at the macro level, there is increased community participation, system-based thinking, and positive community perceptions observed through various community health interventions [33, 36, 46, 54, 60]. Training of community members (i.e., capacity building) and the establishment of support systems have led to improvements in health services and education [55, 61]. Additionally, the introduction of holistic perspectives and tailored motivational approaches enhanced participants’ engagement in health interventions [51]. By advocating for equitable access to healthcare, addressing social inequalities, and promoting community-driven initiatives, relational community engagement has instigated transformative effects on population health and well-being [3, 21, 37, 47].

## Reflections on the implementation of relational community engagement interventions

There is clearly a wide diversity of relational community engagement type interventions. Prioritizing very specific interventions appeared to be challenging with the priority level of intervention (i.e., micro, meso, macro) varying depending on the specific context, population, and health goals. Regardless, tailoring interventions to the local context and need was seen to be key to achieving the desired health outcomes, as well as fostering collective action, social support, and sustainable change. When communities actively participate, they become invested in their own health, often leading to the improved outcomes seen in the body of included articles. Promoting social participation stood out as a key element in the literature due to its consistent positive outcomes with individuals and communities engaged in decision-making processes that fostered a sense of ownership, belonging, empowerment, and accountability.

The review also elucidated some clear evidence gaps in being able to fully understand how and why relational community engagement interventions may contribute to positive health outcomes at all three system levels (micro, meso, macro). For example, previous literature has revealed that community engagement generally may have benefits on micro level health outcomes, including for individuals’ physical and psychological health, as well as their overall psychosocial well-being [62]; however, the literature remains limited on this topic. Previous literature has also shown that community engagement generally can be a valuable approach to improving health outcomes at the meso level; however, reviews summarizing specific interventions is still very much lacking [12]. Interestingly, this review raised an observation that there was a tendency to exclusively report positive outcomes in relevant relational community engagement-type studies. Although, it is possible that mainly positive outcomes came from relational community engagement approaches, it would be helpful to understand if any unreported negative outcomes occurred to better appreciate all the variables involved in relational community engagement. While alertness toward potential bias in reporting studies is important, ignoring all positive outcomes as mere objects of biased reporting would be equally misguided. Regardless, there is substantial need for more comprehensive reporting practices in the area of relational community engagement to ensure a clear understanding of its operational impact.

The COVID-19 pandemic further revealed the importance of community engagement at the macro level [63] highlighting the need for research efforts that have far-reaching implications on broader societal factors. Health systems already engage with diverse communities at multiple levels in settings such as clinics, hospitals, and health posts; through professional and lay roles within essential public health functions (e.g., surveillance); and through accountability mechanisms, such as social participation efforts in policy making and governance [64]. These health system processes are all opportunities to intentionally strengthen connection and trust, and significantly impact the quality of care from the micro to the macro level. Having well-functioning, responsive, trusted, and resilient government systems—particularly health systems—are a mainspring for addressing the biggest crises facing humanity and the planet. Our review highlights that trust can be continuously reinforced or broken in the everyday interactions between service providers and service users across all sectors. The quality and experience of these relationships and services impact the quality of health services, service uptake, and overall health outcomes. With this, our review has identified a few key research streams recommended to build out relational community engagement at the health system level including the following key points:

- Explore and further generate empirical evidence for relational community engagement in the context of healthcare quality and health system performance.
- Build out the understanding for relational interventions and scales of influence, including context-specific country data and metrics.
- Create mechanisms for documenting and responding to the challenges faced during relational community engagement intervention work.
- Develop evidence-based policy options to institutionalize relational community engagement processes within health service design and delivery.

Research agendas for relational community engagement should additionally be underpinned by a social determinants framing, actively embracing complexity, and embodying the developmental nature of human experience. Research efforts should also seek to foreground the social, emotional, and relational processes that remain largely invisible in healthcare practice and medicine as it is practiced today. With this, it will be important to bring together siloed research areas (e.g., brain development, trauma studies, relational sciences), practice, and evidence to identify policy actions that governments can take to strengthen the ‘relational health’ of healthcare systems.

Community-based interventions also often require sustained efforts and resources to achieve long-term impacts. Adequate funding and support are crucial to sustaining relational community engagement initiatives, thereby ensuring their continuous effectiveness. Additionally, tailoring interventions to meet the specific needs and contexts of diverse communities is essential for maximizing their impact and relevance. With this, while studies cover various health issues and settings, it is important to consider the cultural and contextual differences that may influence the outcomes of relational community engagement interventions. A one-size-fits-all approach may not be effective, and interventions will need to be adapted to suit the unique characteristics of each community. Additionally, attention must be paid within future research endeavors to ensure quality metrics are developed and considered within the relational community engagement space. This is to better account for the complexity inherent in relational community engagement interventions that may not be easily categorized to already established quality assessment criteria.

## Concluding reflections

Community engagement has resurfaced in global public health as a critical component in recovery efforts from the COVID-19 pandemic. Yet, there remains ambiguity and a lack of consensus on definitions and scope, with significant gaps in evidence still existing. In addition, advances in scientific understanding posit that the concept of “community” must be broadened to capture the continuum of connection between attachment in early childhood and ongoing processes of social interaction during adolescence and throughout adulthood. “Doing so recognizes that human beings are born, raised, live, play, work, and die in multiple, interconnected communities that continuously shape identity, choices, and behaviors” [65]. Relational community engagement is therefore aligned with the concept of “well-being societies” [66] that emphasizes the responsibility and stewardship of government and other actors through integrated, multi-sectoral responses, from local to national. Our review exploring various relational community engagement interventions revealed this multifaceted impact on micro-, meso-, and macro-level outcomes within community settings. The review highlights the potential versatility and effectiveness of interventions that prioritize relationships, health promotion, and social change while underscoring the significance of holistic and community-centered approaches in addressing diverse health and social challenges.
